# Towards the realistic computer model of precipitation polymerization microgels

**DOI:** 10.1038/s41598-019-49512-3

**Published:** 2019-09-10

**Authors:** Vladimir Yu. Rudyak, Elena Yu. Kozhunova, Alexander V. Chertovich

**Affiliations:** 1 Lomonosov Moscow State University, Faculty of Physics, Moscow, 119991 Russia; 2 Semenov Institute of Chemical Physics, Moscow, 119991 Russia

**Keywords:** Chemical physics, Gels and hydrogels

## Abstract

In this paper we propose a new method of coarse-grained computer simulations of the microgel formation in course of free radical precipitation polymerization. For the first time, we simulate the precipitation polymerization process from a dilute solution of initial components to a final microgel particle with coarse grained molecular dynamics, and compare it to the experimental data. We expect that our simulation studies of PNIPA-like microgels will be able to elucidate the subject of nucleation and growth kinetics and to describe in detail the network topology and structure. Performed computer simulations help to determine the characteristic phases of the growth process and show the necessity of prolongated synthesis for the formation of stable microgel particles. We demonstrate the important role of dangling ends in microgels, which occupy as much as 50% of its molecular mass and have previously unattended influence on the swelling behavior. The verification of the model is made by the comparison of collapse curves and structure factors between simulated and experimental systems, and high quality matching is achieved. This work could help to open new horizons in studies that require the knowledge of detailed and realistic structures of the microgel networks.

## Introduction

Microgels are cross-linked polymer systems of size typically in the range from 100 to 1000 nm. Their unique properties and tunability in terms of architecture, softness, permeability and deformability make them one of the most attractive objects in current soft matter science^1,2^. The importance of microgels for many applications relies on their ability to react to external stimuli (temperature, pH, solution composition, etc), in particular, their swelling/shrinking behavior. Microgels could be used as delivery carriers^3,4^, stimuli-responsive stabilizers of emulsions^5,6^, scavengers^7,8^, photonic crystals^9^, membrane materials^10^ and many other technologies^1,11,12^. *N*-isopropylacrylamide (NIPA) is one of the most widely used monomers for the synthesis of microgel particles, among the few that could be synthesized via surfactant-free process. PNIPA has lower critical solution temperature (LCST) in the region of 32 °C in water, induced by hydrophobic–hydrophilic interactions of NIPA monomers via hydrogen bonding. That allows to use the precipitation polymerization process for the synthesis of PNIPA microgels, in which each microgel particle forms united network during copolymerization of monomers with a small fraction of cross-linker in poor solvent without any additional surfactant^13^. In spite of simplicity of this process implementation, its kinetics is quite complex. The resulting internal structure of microgel particle remains unclear, including network spatial and topological properties.

In order to understand these properties, various computer models of microgels were developed, including atomistic and coarse-grained. Atomistic models are the invaluable tool for the exploration of molecular mechanisms driving microgels behaviour^14–19^, but the drawback is that they are too limited in spatial and time scales to gain the insight on the behaviour of a microgel particle as a whole. The variety of coarse-grained models help to study more general properties of microgel as a single particle. In the simplest and most popular coarse-grained model microgels are constructed from crystalline lattice (typically diamond one) by replacing each crystal edge with a subchain and each node with a cross-linker^20,21^. The system is then cut out in form of a sphere or ellipsoid to obtain a particle of finite size. This approach allows to study various general phenomena such as collapse and uptake-release dynamics of hollow and layered particles^4,22^, behavior of interpenetrating networks (IPN)^23^, and microgels at the liquid–liquid interface^5,8^ and other effects^24^. At the same time, crystalline lattice model has multiple limitations, including unrealistic subchain length distribution, major underestimation of dangling ends number, uniform distribution of cross-linkers, the absence of network defects and entanglement between subchains^25^. All these factors affect the behaviour of the model and limit its application cases.

More sophisticated models were developed to overcome these limitations. Nikolov *et al*.^26^ prepared microgel particles by randomly distributing cross-linkers within a cubic simulation box and then connecting neighbour cross-linker particles by subchains. In another work, functionalized polymer chains were cross-linked in a spherical confinement^27^, simulating the emulsion polymerization process. Unfortunately, that approach could not be applied for modeling of typical surfactant-free polymerization of PNIPA-like microgels and other common compounds due to the cardinal difference in reaction mechanisms. Gnan *et al*.^28^ obtained random microgels by self-assembly of patchy particles mimicking the polymerization process of PNIPA microgels as step-growth polymerization. The resulting structures showed more realistic properties compared to regular lattice model^29^. Although it should be noted that the polymer network formation mechanism in this model differs from the precipitation polymerization mechanism of PNIPA microgels. First, the network of patchy particles forms by step-growth mechanism, thus the polymerization kinetics is completely unlike typical radical polymerization process. Second, the process itself does not allow to introduce the initiator molecules and to consider dangling ends in the network topology. Finally, this model does not reproduce high sensitivity of real precipitation polymerization process to concentrations of initial components and solvent quality due to the spherical confinement applied to the reagents. The kinetics of microgel formation in these models can be additionally compared with kinetic models^30,31^. More details on the comparison of various microgels simulation models can be found in review^25^.

All in all, the described models work well for the generation of random microgel structures and allow to check the results obtained by crystalline lattice models for the defects caused by its regularity. At the same time, there are many complex effects in microgels that are respectively more sensitive to the precise inner structure of the network. That said, new frontiers in microgels exploration require the knowledge of detailed and realistic structures of the microgel networks. For example, the study of microphase separation inside microgels or of the self-organized core-shell structures in swollen microgels is impossible without the consideration of spatial correlations of subchains length distribution, entanglements and dandling ends. Also, the *in silico* synthesis of more sophisticated structures, such as compolymer and block-copolymer microgels, interpenetrating network microgels is required for more reliable results.

In answer to that demand, in this paper we formulated the method of coarse-grained simulations of the realistic precipitation polymerization formation of PNIPA-like microgel particles. We discussed the features of the curing process, analyzed the resulting structures and compared them to dynamic light scattering (DLS), static light scattering (SLS) and small-angle X-ray scattering (SAXS) data on PNIPA microgels.

## Results

### Simulations of precipitation polymerization process

We prepared the realistic structures of PNIPA-like microgels by simulating the free-radical precipitation polymerization process. We implemented “mesoscale chemistry” concept into the framework of coarse-grained (CG) molecular dynamics method. For this purpose, we prepared the solution of initial components: monomer (M), cross-linker (L) and initiator (I). Each M, L and I molecule was presented as a single CG particle. The mass ratio of components M:L:I was 98.5:1.0:0.5, which corresponds to ratios of chemicals used in laboratory synthesis (see Methods). Simulation box was randomly filled with particles up to total mass density equal to 2.4%. The implicit solvent model in NVT ensemble was used (i.e. solvent was presented via the interactions between M/L/I particles instead of addition of the specific solvent particles). “Poor” quality of the implicit solvent was set by ${R}_{LJ}^{{\rm{cut}}}=1.4$ (see Methods for the detailed information on the CG model, interactions and ensemble). Then each CG particle was assigned with valence, defining the maximum number of bonds it can form: two for monomer particles M, four for cross-linker particles L, and one for initiator particles I. Additionally, active center marks were assigned to initiator particles.

During the simulation of polymerization process the following chemical reactions were allowed:

1. **Initialization reaction**: I^*^ + ⋅M⋅ → I − M^*^, when an initiator particle with a free valence and active center mark (I^*^) and monomer particle with a free valence and no active center mark (⋅M⋅) form a new bond. The active center mark is passed from the initiator to the monomer particle.

2. **Monomer–monomer addition reaction**: M^*^ + ⋅M⋅ → M − M^*^, when a monomer particle with a free valence and active center mark (M^*^) and monomer particle with a free valence and no active center mark (⋅M⋅) form a new bond. The active center mark is passed to the second monomer particle.

3–4. **Monomer–cross-linker addition reactions**: M^*^ + :L: → M − :L^*^ and M^*^ + L: → M − L^*^, when a monomer particle with a free valence and active center mark (M^*^) and cross-linker particle with a free valence and no active center mark (:L: or L:) form a new bond. The active center mark is passed from the monomer to the cross-linker particle.

5–6. **Cross-linker–monomer addition reactions**: :L^*^ + ⋅M⋅ → :L − M^*^ and L^*^ + ⋅M⋅ → L − M^*^, when a cross-linker particle with a free valence and active center mark (:L^*^ or L^*^) and monomer particle with a free valence and no active center mark (⋅M⋅) form a new bond. The active center mark is passed from the cross-linker to the monomer particle.

According to work^32^ the cross-linker–cross-linker reaction rate (BIS–BIS) is lower by two orders of magnitude than the normal propagation steps, both monomer–monomer and monomer–cross-linker, thus this reaction was neglected in our model.

These reactions were implemented probabilistically. At regular time intervals (200 steps), the distance between an active particle and adjacent particles with free valencies was compared to the reaction radius *R*_react_ = 1. A new bond was formed with the probability *p*_react_. We started from value of *p*_react_ small enough to keep quasi-equilibrium conditions in a vicinity of reaction centers, and increased it as the concentration of free monomers decreased. The following protocol was used:

1. Equilibrate the initial mixture for 1 × 10^5^ CG MD steps (chemical reactions are off).
2. Set chemical reactions on, *p*_react_ = 7 × 10^−4^ and run 3 × 10^8^ CG MD steps.
3. Set *p*_react_ = 7 × 10^−3^ and run 2 × 10^8^ CG MD steps.
4. Set *p*_react_ = 7 × 10^−2^ and run 2 × 10^8^ CG MD steps.

For all systems, that protocol resulted in structures formation with conversion rate $c={N}_{b}/{N}_{b}^{{\rm{\max }}} > 0.999$, where *N*_*b*_ is a number of bonds created during polymerization process, ${N}_{b}^{{\rm{\max }}}$ is a maximum possible number of bonds in the system.

All simulations were performed in LAMMPS package^33^ with modified fix_bond_create module which implements multiple reactions. Modified LAMMPS module, run script sample and resulting synthesized 39000-particle microgel structure (with sol fraction cut out) are available for download^34^.

### Microgel formation kinetics

**Figure 1:**
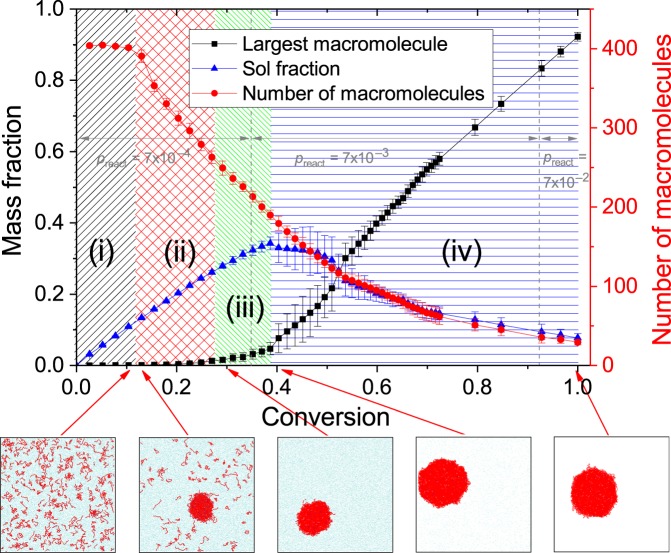
The largest macromolecule mass fraction, sol mass fraction and total number of macromolecules depending on the conversion. Inserts show snapshots of a system at various stages of polymerization. Monomer and cross-linker particles are shown in cyan (single particles) and red (in polymer chains); initiator particles are shown in blue.

We studied in detail the kinetics of microgel formation using the model described above. During the polymerization process, we observed the changes of the following parameters. Conversion *c* was calculated as a ratio between the number of created bonds and maximum possible number of bonds in the system. The mass of the largest macromolecule, the number and total mass of other macromolecules were calculated by topological analysis. Figure 1 shows the dependency of these properties on the conversion and the characteristic system snapshots at various stages. The data were averaged by 10 independent runs, error bars show the standard deviation.

At the initial setup, the components (monomer, initiator, cross-linker) are completely soluble at any concentrations, but growing chains collapse at some degree of polymerization, which is the main feature of precipitation polymerization. This was achieved by the use of the implicit solvent model with the solvent quality set to “poor” by cut-off radius ${R}_{LJ}^{{\rm{cut}}}=1.4$, corresponding to Flory-Huggins parameter *χ* > 1/2 (see Methods and Collapse properties). The whole polymerization process can be divided into four phases: (i) homogeneous polymerization and formation of a single nucleus, (ii) collapse of all chains onto the nucleus, (iii) growth of the nucleus and individual chains collapsed on the nucleus, and (iv) rapid growth of the nucleus due to intensive merging with other macromolecules and following polymerization up to *c* → 1.

Phase (i) lasts up to conversion of 0.11–0.12, according to our simulations. In this phase, chains are too short to collapse, thus they grow independently, which is confirmed by almost constant number of polymer chains in the system. Phase (i) ends at the point when (some) chains become long enough to collapse due to “poor” solvent quality. Then, eventually, some of these molecules cross-link with other ones and start the collapse process. The number of macromolecules begins to decay from this moment, as seen on Fig. 1. It should be noted that multiple nuclei could be found in some realizations up to *c* < 0.15. However, in all the systems observed the single nucleus was formed at *c* = 0.15. Phase (i) takes approximately 2.4 × 10^5^ time units (80 million steps), which roughly corresponds to 2–3 minutes of real synthesis process (see Methods).

Phases (ii) and (iii) are characterized by gradual growth of all macromolecules and simultaneous slow merging of individual chains. The primary difference between these phases is shown on inset snapshots of Fig. 1: when the conversion value exceeds 0.27–0.28 all the macromolecules collapse onto the single spherical-like particle, which signifies the beginning of phase (iii). Phases (ii) and (iii) take totally 9 × 10^5^ time units (300 million steps), which roughly corresponds to 9–13 minutes of real synthesis process.

During phase (iv), the largest macromolecule rapidly grows by adsorbing and cross-linking with other long chains: the largest macromolecule size (black curve on Fig. 1) rapidly increases, while the overall mass of other macromolecules (blue curve) declines. After *c* ≈ 0.5, the initial rapid growth gives way to the final stage of polymerization, during which the microgel particle grows by adsorbing the remaining single units and merging with other still growing polymer chains. At *c* = 1, the microgel particle reaches the 92% ± 1% of the overall mass, and the left 8% ± 1% consist of 30 ± 4 macromolecules with molecular masses of 220 ± 80, so-called sol-fraction. This last phase takes near half of simulation time (300 more million steps), which corresponds to near 5–7 hours of real synthesis process.

We found several important features of the polymerization process which are almost impossible to capture experimentally due to very fast passing of the first three phases. First, the collapse of the chain on the nucleus during phases (i) and (ii) does not lead to its immediate merging with the nucleus by cross-linking. At the end of phase (ii), when all the chains are collapsed altogether, the mass of the largest molecule is just 5% of the total mass of macromolecules. And at the end of phase (iii), this value grows only up to 12%. Their further merging into a single microgel particle occupies a very long period of time (50% of simulation time, or near 98% of real synthesis time). Second, there is no significant increase of either reagents concentration or reaction speed after the nucleus formation. Microgel particle grows gradually during the diffusion of the initial components from the solution. The reasons are purely entropic: it is too unfavorable for the single particles to be absorbed, even in the solvent of poor quality. These results directly demonstrate that the microgels particle synthesis should not be considered finished when the solution turns opaque – that indicates the formation of nuclei on phase (ii). Thus the synthesis should be continued for several more hours to obtain stable microgel particles.

### Collapse properties

**Figure 2:**
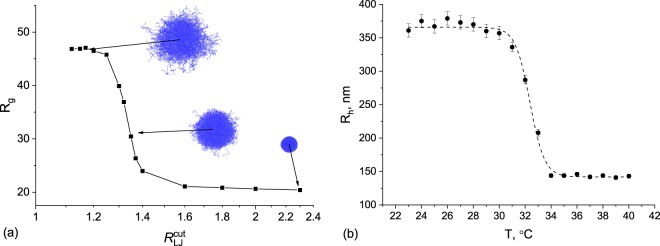
(**a**) Gyration radius *R*_*g*_ as a function of a ${R}_{LJ}^{{\rm{cut}}}$ obtained in computer simulation. Also typical snapshots are presented. (**b**) Experimental dependence of hydrodynamic radius *R*_*h*_ on the temperature for PNIPA microgel obtained by DLS method.

To verify a computer model, one needs to compare the obtained *in silico* microgels with experimental systems. One of the most impressive features of polymer chains is their ability to change the conformational state from coil to globule depending on the solvent quality, which brings the variety of stimuli-responsive applications. Hence, the analysis of microgel behavior near the coil–globule transition point (so-called *θ*-point) of its subchains could be a good benchmark for the comparison of simulations and experimental data. In our computer modeling, the solvent quality was controlled by the Lennard-Jones potential cut-off ${R}_{LJ}^{{\rm{cut}}}$, and the microgel collapse was observed by the changes in radius of gyration *R*_*g*_ (Fig. 2a). In experimental realization, we studied the behavior of PNIPA microgels near LCST point DLS method, thus the solvent quality was controlled by its temperature and the collapse was observed by the changes in hydrodynamic radius *R*_*h*_ (Fig. 2b). Both collapse curves show the similar dependence of microgel size on solvent quality with the sharp transition from swollen to shrunken state. The swelling ratios are of the same magnitude for both systems (2.3 in simulations and 2.5 in the experiment), although we should compare *R*_*g*_ to *R*_*h*_ only qualitatively for such soft objects.

**Figure 3:**
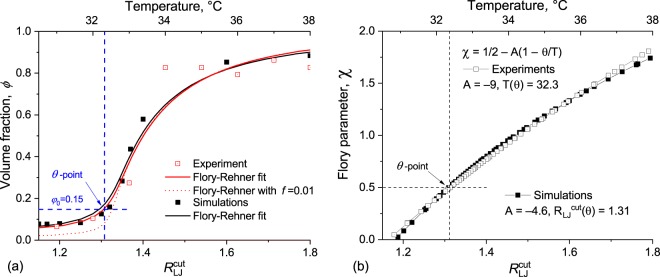
(**a**) Superposition of experimental and simulated data of microgel volume fraction *ϕ* and fitting curves applying Flory-Rehner theory; (**b**) Matching of *χ*(*T*) and $\chi ({R}_{LJ}^{{\rm{cut}}})$ for correspondence between experiment and simulation parameters.

At the same time, it is possible to establish the quantitative matching between cut-off radius ${R}_{LJ}^{{\rm{cut}}}$, microgel polymer volume fraction *ϕ* and Flory parameter *χ* by application of the well-known Flory-Rehner model^35^ to the data (fitting details are described in Methods section, note that the only variable parameters for experimental data set were *θ*-temperature and cross-linker fraction *f*, while in simulations we additionally varied model-dependent parameter *A*). The results are shown in Fig. 3. It could be seen that the dependencies of polymer volume fraction on solvent quality in the vicinity of *θ*-point fit very closely for both computer simulation and experimental data. With some limitations, the Flory-Rehner theory allows us to directly correlate simulation and experimental parameters. By matching the *θ*-point on the dependencies of *χ*(*T*) and $\chi ({R}_{LJ}^{{\rm{cut}}})$ one can get an exact correspondence between *T* and ${R}_{LJ}^{{\rm{cut}}}$, see Fig. 3b. In future, this can help to choose the simulation parameters more accurately.

More details should be said about the effective density of cross-links, which also could be estimated with the use of Flory-Rehner model. In our setup, the initial amount of cross-links was 1%. The theoretical curve, corresponding to that value, is shown on Fig. 3a as dotted line. One could see that this curve is too sharp and the resulting swelling ratio is too high for a realistic system, and does not fit neither experimental data nor computer simulations results. To obtain better fitting we had to use a model with higher cross-link density values *f*. Partially it could be explained by the network topology. Our computer simulations directly show for the first time that around half of the microgel molecular weight is attributed to long elastically inactive dandling ends (see below). Thus the elastically active network consists of shorter subchains which results in higher effective cross-linking density. For *in silico* microgels, the amount of effective cross-links grows from 1% to 1.6%. In real synthesis, additional cross-links also appear due to different side reactions^36^. To summarize, the cross-linking density *f* in Flory-Rehner model is an effective value and it is not correct to set that value directly equal to cross-linker concentration in the initial solution, both in simulations and experiments.

### Microgel structure and topology

Different scattering methods, such as SLS, SANS and SAXS, allow us to vaguely estimate the detailed structure of a real microgel particle by comparison of obtained structure factor to theoretical models of radial density distribution. Such analysis works quite well for the systems with simple geometry and narrow particle size distribution, i.e. PNIPA microgels. The best and relatively easy fitting could be achieved by the use of so-called Fuzzy Sphere Model^37^, where the particle is represented as a core with constant density followed by a corona which density decreases sigmoidally.

**Figure 4:**
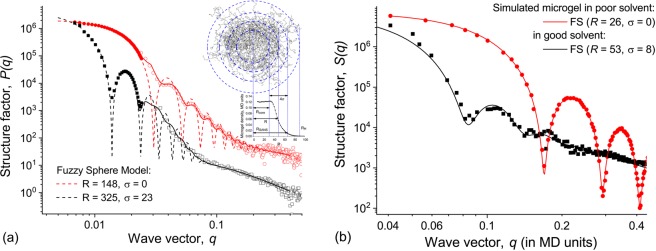
(**a**) Experimental scattering data for PNIPA microgels obtained by SLS and SAXS: collapsed state (*T* = 50 °C, red circles), swollen state (*T* = 23 °C, black squares). Lines of corresponding colors present the structure factors for Fuzzy Sphere Model with given parameters (dashed) and its smoothing (solid). The inset illustrates the corresponding internal structure. (**b**) Structure factors of the simulated microgels (dots) and fitting by Fuzzy Sphere Model (solid lines) in different solvent conditions: “good” solvent (${R}_{LJ}^{{\rm{cut}}}=1.12$, black) and “poor” solvent (${R}_{LJ}^{{\rm{cut}}}=2.3$, red).

Figure 4a represents experimental structure factors of PNIPA microgels in two conformational states: swollen in “good” solvent (below PNIPA LCST, black squares) and collapsed in “poor” solvent (above PNIPA LCST, red circles). The data were collected using both SLS and SAXS measurements for broader *q* values coverage. These data could be very well fitted by Fuzzy Sphere model curves with appropriate smoothing (black and red lines, respectively). By this, we estimated both the radius of the microgel particle (*R* ≈ 325 nm and *R* ≈ 150 nm in swollen and collapsed states, correspondingly) and the size of the less dense corona (*σ* ≈ 23 nm and *σ* ≈ 0 nm in swollen and collapsed states, correspondingly).

To compare these data with the results of computer simulations, we calculated structure factors (see Fig. 4b) of simulated microgels in “good” (${R}_{LJ}^{{\rm{cut}}}=1.12$) and “poor” (${R}_{LJ}^{{\rm{cut}}}=2.3$) solvents. The simulated structure factor curves could also be fitted well by Fuzzy Sphere model, and the resulting parameters match the density profile data. Overall, the fittings for experimental data and simulation results are in good agreement, as well as ratios between *R* and *σ* in swollen and collapsed states. Taking it into consideration, it is important to note how complicated it is to perform the fitting of experimental structure factors of more sophisticated systems (IPN microgels, copolymer microgels, block-copolymer microgels, etc.) by using only theoretical models. Due to increasing polydispersity of the particles and unclear internal structure an ambiguous interpretation of the data is possible in these systems. From this point of view, computer simulations open up opportunities to decipher, analyze and visualize the obtained experimental systems.

**Figure 5:**
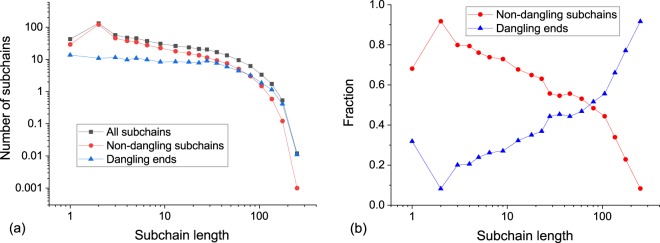
(**a**) Subchain length distributions for all subchains, non-dangling subchains and dangling ends. (**b**) Fractions of non-dangling chains and dangling ends. All data were averaged by 10 independent realizations.

We also produced the topological analysis of *in silico* microgel particles. For that we synthesized 10 independent structures. The resulting systems were cleared from sol fraction and analyzed. Figure 5a shows the subchain length distribution in double-log scale. It should be noted that the plotted values are distribution densities instead of histograms. Generally, the distribution is close to exponential, but with fast decay in subchains longer than 80–100 beads. For deeper understanding, we divided the whole structure into two parts: dangling ends and the main network. We defined dangling ends as subchains with one free end which is not chemically bonded to any other subchain. To our surprise, we found that dangling ends form approximately 50% ± 1% of the total microgel particle mass in our simulations. Interestingly, dangling ends and the main network contribute differently to the statistics of subchain length. Average subchain length was 31.4 ± 0.5 for the main network, and 51.1 ± 0.7 for dangling ends. Thus, the dangling ends are in average longer than elastically active subchains, and also are distributed more equally around various lengths (its distribution is almost horizontal between 1 and 40 unit length). In sum, the short subchains are mostly ones in the main elastic network, while the long chains are mostly dangling ends (see Fig. 5b). That influences the overall collapse-swelling and mechanical properties of such microgels.

**Figure 6:**
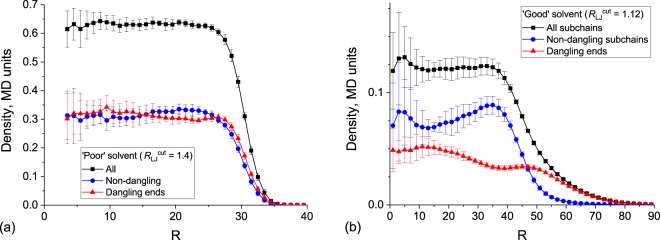
Density profiles of all components, non-dangling subchains and dangling ends in (**a**) ‘poor’ solvent (${R}_{LJ}^{cut}=1.4$) and (**b**) ‘good’ solvent (${R}_{LJ}^{cut}=1.12$). Average values and error bars were calculated from 10 independent realizations.

Finally, we studied in detail the density distributions in simulated microgels. Averaged radial density plots right after the synthesis (in collapsed state) and after the equilibration in ‘good’ solvent are shown on Fig. 6. Generally, microgel particles in good solvent have plain density distribution in the central part and loose corona with gradual density decrease. We analyzed the contributions of dangling ends and elastic network in density profiles (see red and blue lines in Fig. 6, correspondingly). While these two parts contribute equally to the density profile in the collapsed state, their relative distribution changes significantly in the swollen state. The dangling ends can easily move form the microgel bulk volume into the loose corona, leading to the enrichment of the core with the elastic network (especially its border region). It brings us to the conclusion that the well-defined corona of swollen PNIPA-type microgel consists mainly of dangling ends.

The swollen PNIPA microgel particles obtained in our experiments were about 330 nm in radius. The radius of the simulated particles in the swollen state was about *R* ≈ 75 MD units, which is roughly 40 nm. In this viewpoint the question of corona scaling reasonably appears. To clarify that matter, we performed the computer simulations of microgels with various molecular masses, see Figure S4 in SI. We found that starting from the molecular mass about 10^4^ the corona profile and its absolute width is almost stable and do not change upon further molecular mass increase. That means that the proportion of corona size in the swollen microgel would rapidly decrease with the increase of microgel molecular weight.

## Conclusions

In this paper, we performed *in silico* investigations of microgel precipitation polymerization, and compared the obtained results with experimental data on PNIPA microgels. This analysis led us to several important conclusions:

1. The process of precipitation polymerization of a microgel can be divided into four distinct phases: (i) homogeneous polymerization of individual chains and formation of the single nucleus, (ii) collapse of all polymer chains onto the nucleus, (iii) growth of the sol fraction inside the nucleus, and (iv) cross-linking of sol molecules into single network. The united microgel particle is formed only during phase (iv) (after 40% conversion) and takes most of the time of synthesis process.
2. A significant portion of the microgel molecular weight is occupied by the dangling ends, which in our case (1% of the cross-linker and 0.5% of the initiator) is half of the total mass of the microgel. Moreover, dangling ends are in average noticeably longer than subchains belonging to the elastically active network. The presence of dangling ends effectively increases the concentration of cross-linker in elastically active network followed by the decrease of swelling ratio in comparison to the theoretical expectations.
3. The density profile and structure factor of swollen microgel is well described by the Fuzzy Sphere Model, which loose corona is formed mainly by the dangling ends. For large systems the corona density profile and its width do not significantly depend on the size of the microgel.

In general, our model allows to reproduce the precipitation polymerization synthesis of microgel particles of a size close to the real systems with state-of-art degree of detail. Further development of our model should take into account the following possible drawbacks: instant initiation (i.e. all initiators are activated simultaneously), neglect of termination processes (namely recombination and disproportionation), and electric neutrality of the system.

## Methods

### Experimental

Monomer N-isporopylacrylamide (NIPA, Wako, Japan), cross-linker N, N′-methylenebisacryl-amide (BIS, Fluka, Switzerland) and initiator potassium persulfate (PS, Wako, Japan) were used as received. Water was purified using a Milli-Q system (Millipore, USA). Thermosensitive microgels were synthesized by free-radical thermo-initiated precipitation polymerization in water in the presence of BIS cross-linker (NIPA concentration in water - 0.01 g/ml, BIS - 1 molar percent to NIPA, PS - 3 molar percent to NIPA). Detailed description of the synthetic procedure could be found in our experimental paper^38^.

SLS and DLS measurements were performed by a static/dynamic compact goniometer (DLS/SLS-5000, ALV, Langen, Germany). A HeNe laser with a power of 22 mW emitting a polarized light at *λ* = 6328 Å was used as the incident beam. SLS intensity functions were taken at 23 and 50 °C at scattering angles of 30 to 150°. The studies of temperature dependencies were carried out at a scattering angle of 90°, the heating rate was 1 °C/min, at each temperature the samples were held for 15 minutes before the measurements.

X-ray synchrotron radiation small-angle scattering data were collected at the BioSAXS station (Kurchatov synchrotron radiation source) with energy of incident beam 8 keV (*λ* = 1.445 Å) using a 2D DECTRIS Pilatus3 1M photon-counting detector. Beam was collimated using a system of four slits and focused on the detector by monochromator in horizontal dimension and by mirror in vertical dimension. Size of the beam on the detector was approximately 0.5 × 0.35 mm. Preliminary instrument calibration was performed just before the experiments using a silver behenate sample with an exposure of 60 seconds. Sample-to-detector distance was 2500 mm that provides a scattering vector range from 0.003 to 1.5 nm^−1^. Microgel dispersion samples were placed in a cylindrical capillaries 5 cm length and 2 mm in diameter. Measurements were performed at two different temperatures: 25 °C and 50 °C. The data was normalized to the intensity of the transmitted beam and radially averaged. The scattering of the solvent (distilled water) was subtracted and the curves were scaled using the PRIMUS program from ATSAS software suite.

### Coarse-grained model

In our simulations of microgels, we used a coarse-grained (CG) model with implicit solvent. Monomer, cross-linker and initiator molecules were represented as coarse-grained particles (also called “beads”) with unit mass. Microgel network structure was determined by bonds between the particles. For all bonds, we used FENE potential^39^ with bond strength *K*_*b*_ = 30, equilibrium bond length *R*_*b*_ = 1.5, and LJ parameter *ε*_*b*_ = 0. Harmonic angle potential with angle strength *K*_*a*_ = 1 and equilibrium angle *θ*_*a*_ = 180° was used. Cutoff 12/6 Lennard-Jones potential with *ε*_*LJ*_ = 2 and *σ*_*LJ*_ = 1 was applied to all pairs of particles. The value of LJ cutoff radius ${R}_{LJ}^{{\rm{cut}}}$ was used to define the implicit solvent quality: lower values corresponded to “good” solvent, and higher values corresponded to “poor” solvent (see Fig. 3 for ${R}_{LJ}^{{\rm{cut}}}$–*χ* mapping). Coarse-grained molecular dynamics simulations in canonical ensemble were performed. Nose-Hoover thermostat with temperature *T* = 1 with temperature damping time *τ*_dump_ = 50 was used.

### Time scale of *in silico* polymerization

The overall experimental synthesis composition to compare with simulation parameters was monomer:cross-linker:initiator = 100:1:6. According to Polymer Handbook^40^ the PS initiator half-life period is about 3 hours at 80 °C, thus we suppose that only about 10% of initiator was activated during initial nucleus formation. Time required for chain propagation by one monomer unit could be estimated as 1 ms using propagation rate constant for acrylic monomers^40^, and 10% of conversion (the time when single nucleus is formed) is reached in about few minutes in real experiment. It allows to roughly estimate the effective time scale in simulations by taking into consideration both simulation time and current reaction probability. The lengths of different phases of the synthesis process are the following: (i) 2.4 × 10^5^ MD time units for 0 to 10% conversion, 2–3 minutes of real-time process; (ii) 3.6  ×  10^5^ MD time units for 10% to 28% conversion, 4–6 minutes of real-time process; (iii) 1.9 × 10^5^ MD time units for 28% to 40% conversion, 5–8 minutes of real-time process, (iv) 9 × 10^5^ MD time units for 40% to 100% conversion, 5–7 hours of real-time process. Estimations of real-time lengths of phases (iii) and (iv) are disproportionally larger compared to phases (i) and (ii) due to correction by higher reaction probability *p*_react_.

### Equilibration in various solvent quality conditions

We studied collapse kinetics of the prepared systems. First, we removed sol fraction and produced subsequent analysis only for the largest particle in each system (which is 92% ± 1% of initial mass). Next, we set desired solvent quality by adjusting ${R}_{LJ}^{{\rm{cut}}}$ and equilibrate the system for 3 × 10^7^ steps (equal to 90000 MD time units). Then we use following 5 × 10^6^ steps to generate density plot, radius of gyration *R*_*g*_ and structure factor *S*(*q*).

### Structure factor calculations

In addition to snapshots observation the morphology of the system at each point could be thoroughly analyzed by calculating the static structure factor^41^ as $S(q)=\frac{1}{n}\mathop{\sum }\limits_{j=1}^{n}\exp {(iq{r}_{j})}^{2}$, where *n* is the total number of particles and averaging is performed over the wave vector set {*q*_*x*_, *q*_*y*_, *q*_*z*_} = {2*πk*/*l*_*x*_, 2*πm*/*l*_*y*_, 2*πp*/*l*_*z*_}, where *k*, *m*, *p* are integers from 1 to 32, and over a sequence of independent system conformations. As a rule, 50 conformations separated by 10000 MD steps were taken for the averaging. This static structure factor is directly proportional to the intensity of some scattered beam measured in light, X-ray, electron or neutron elastic scattering experiments. Thus it is the characteristic which could be directly compared with experimental data from SLS and SAXS.

To compare structure factor with some model and with experiment data from SLS and SAXS we used well known Fuzzy Sphere model, initially proposed in^37^.

### Flory-Rehner fitting

To understand the collapse behavior and compare experimental and simulation results we follow the Flory approach of coil to globule transition^42^, which estimates the Flory-Huggins parameter *χ* near theta-point:

1 $$\chi (T)=1/2-A(1-\theta /T).$$

This equation could be coupled with the Flory-Rehner model^35^ of equilibrium gel swelling as a balance between mixing contribution and elastic stretching:

2 $$\chi (\varphi )=\frac{1}{{\varphi }^{2}}\{\frac{{N}_{C}{\nu }_{S}}{{V}_{0}{N}_{A}}[\frac{\varphi }{2{\varphi }_{0}}-{(\frac{\varphi }{{\varphi }_{0}})}^{1/3}]-\varphi -\,\mathrm{ln}(1-\varphi )\}.$$

These equations give the implicit collapse curve of any polymer gel in a close vicinity of theta-point. We take *A* = −9 to fit experimental data as an average value for a number of literature data as presented in^35^. Also following Lopez *et al*. (see supplementary in^35^) we fix prefactor $\frac{{N}_{C}{\nu }_{S}}{{V}_{0}{N}_{A}}$ as 0.35*ϕ*_0_ *f* for experimental setup with PNIPA and 2.0*ϕ*_0_ *f* for simulation data (*f* is the cross-link density). We aware that the form of *χ*(*T*) dependence is oversimplified and valid as a zero approximation only in a close vicinity of *θ*-point, where *ϕ* = *ϕ*_0_. Additional terms as *χ* = *χ*(*A*) + *Cϕ* + *Dϕ*^2^ + ... could be used to more precise fitting, but it works only outside the *θ*-point region and does not change significantly the transition curve near *θ*-point. The fitting procedure itself was performed in Origin^43^ by Implicit Curve Fit function, see function file Flory-Rehner.fdf in package^34^.

To recalculate hydrodynamic (from DLS measurements) and gyration (from simulations) radii into volume density we use the simple relation *ϕ*/*ϕ*_*c*_ = (*R*_*c*_/*R*)^3^, where *ϕ*_*c*_ and *R*_*c*_ are the volume fraction and radius in a collapsed state, correspondingly. Also we use the value of the volume fraction in the collapsed state equal to 0.8 for the experimental data^35^.

## Supplementary information

Supporting Information Towards the realistic computer model of precipitation polymerization microgels

## Data Availability

The datasets generated and analysed during the current study are available from the corresponding author on reasonable request.

## References

[1] Plamper FA, Richtering W (2017). Functional microgels and microgel systems. Accounts Chemical Research.

[2] Lu Yan, Ballauff Matthias (2011). Thermosensitive core–shell microgels: From colloidal model systems to nanoreactors. Progress in Polymer Science.

[3] Richtering W, Potemkin II, Rudov AA, Sellge G, Trautwein C (2016). Could multiresponsive hollow shell–shell nanocontainers offer an improved strategy for drug delivery?. Nanomedicine.

[4] Schmid AJ (2016). Multi-shell hollow nanogels with responsive shell permeability. Scientific Reports.

[5] Geisel K, Rudov AA, Potemkin II, Richtering W (2015). Hollow and core–shell microgels at oil–water interfaces: Spreading of soft particles reduces the compressibility of the monolayer. Langmuir.

[6] Liu T (2012). Non-coalescence of oppositely charged droplets in ph-sensitive emulsions. Proceedings National Academy Sciences.

[7] Gumerov RA (2016). Mixing of two immiscible liquids within the polymer microgel adsorbed at their interface. ACS Macro Letters.

[8] Rumyantsev AM, Gumerov RA, Potemkin II (2016). A polymer microgel at a liquid-liquid interface: theory vs. computer simulations. Soft Matter.

[9] Lyon LA (2004). Microgel colloidal crystals. The Journal Physical Chemistry B.

[10] Lohaus T (2017). Tunable permeability and selectivity: Heatable inorganic porous hollow fiber membrane with a thermoresponsive microgel coating. Journal Membrane Science.

[11] Mei Y, Lu Y, Polzer F, Ballauff M, Drechsler M (2007). Catalytic activity of palladium nanoparticles encapsulated in spherical polyelectrolyte brushes and core-shell microgels. Chemistry Materials.

[12] Saunders BR (2009). Microgels: From responsive polymer colloids to biomaterials. Advances Colloid Interface Science.

[13] Pich, A. & Richtering, W. (eds) *Chemical Design of Responsive Microgels* (Springer Berlin Heidelberg, Berlin, Heidelberg, 2011).

[14] Deshmukh Sanket A., Sankaranarayanan Subramanian K. R. S., Suthar Kamlesh, Mancini Derrick C. (2012). Role of Solvation Dynamics and Local Ordering of Water in Inducing Conformational Transitions in Poly(N-isopropylacrylamide) Oligomers through the LCST. The Journal of Physical Chemistry B.

[15] Kang Y, Joo H, Kim JS (2016). Collapse–Swelling Transitions of a Thermoresponsive, Single Poly(*N* - isopropylacrylamide) Chain in Water. The Journal Physical Chemistry B.

[16] Bo.tan V, Ustach V, Faller R, Leonhard K (2016). Direct Phase Equilibrium Simulations of NIPAM Oligomers in Water. The Journal Physical Chemistry B.

[17] Mukherji, D., Marques, C. M., Stuehn, T. & Kremer, K. Depleted depletion drives polymer swelling in poor solvent mixtures. Nature Communications **8**, 10.1038/s41467-017-01520-5 (2017). 10.1038/s41467-017-01520-5 PMC5680348 29123108

[18] García EJ, Bhandary D, Horsch MT, Hasse H (2018). A molecular dynamics simulation scenario for studying solvent-mediated interactions of polymers and application to thermoresponse of poly(N-isopropylacrylamide) in water. Journal Molecular Liquids.

[19] García EJ, Hasse H (2019). Studying equilibria of polymers in solution by direct molecular dynamics simulations: poly(Nisopropylacrylamide) in water as a test case. The European Physical Journal Special Topics.

[20] Claudio GC, Kremer K, Holm C (2009). Comparison of a hydrogel model to the poisson–boltzmann cell model. The Journal Chemical Physics.

[21] Kobayashi H, Winkler RG (2014). Structure of microgels with debye–hückel interactions. Polymers.

[22] Masoud Hassan, Alexeev Alexander (2011). Controlled Release of Nanoparticles and Macromolecules from Responsive Microgel Capsules. ACS Nano.

[23] Rudyak VY, Gavrilov AA, Kozhunova EY, Chertovich AV (2018). Shell–corona microgels from double interpenetrating networks. Soft Matter.

[24] Schroeder R (2015). Electrostatic interactions and osmotic pressure of counterions control the ph-dependent swelling and collapse of polyampholyte microgels with random distribution of ionizable groups. Macromolecules.

[25] Rovigatti L, Gnan N, Tavagnacco L, Moreno AJ, Zaccarelli E (2019). Numerical modelling of non-ionic microgels: an overview. Soft Matter.

[26] Nikolov S, Fernandez-Nieves A, Alexeev A (2018). Mesoscale modeling of microgel mechanics and kinetics through the swelling transition. Applied Mathematics Mechanics.

[27] Moreno AJ, Lo Verso F (2018). Computational investigation of microgels: synthesis and effect of the microstructure on the deswelling behavior. Soft Matter.

[28] Gnan N, Rovigatti L, Bergman M, Zaccarelli E (2017). In silico synthesis of microgel particles. Macromolecules.

[29] Rovigatti L, Gnan N, Zaccarelli E (2017). Internal structure and swelling behaviour of in silico microgel particles. Journal Physics: Condensed Matter.

[30] Janssen FAL (2017). Synthesis of Poly(*N* -vinylcaprolactam)-BasedMicrogels by Precipitation Polymerization: Process Modeling and Experimental Validation. Industrial & Engineering Chemistry Research.

[31] Janssen Franca A.L., Ksiazkiewicz Agnieszka, Kather Michael, Kröger Leif C., Mhamdi Adel, Leonhard Kai, Pich Andrij, Mitsos Alexander (2018). Kinetic Modeling of Precipitation Terpolymerization for Functional Microgels. Computer Aided Chemical Engineering.

[32] Kröger Leif C., Kopp Wassja A., Leonhard Kai (2017). Prediction of Chain Propagation Rate Constants of Polymerization Reactions in Aqueous NIPAM/BIS and VCL/BIS Systems. The Journal of Physical Chemistry B.

[33] Plimpton S (1995). Fast parallel algorithms for short-range molecular dynamics. J. Comp. Phys..

[34] LAMMPS module for multiple reactions with samples, Laboratory of Microstructured Polymer Systems. http://polly.phys.msu.ru/~rudyak/lammps_fix_bond_create.html. Accessed: 2019-02-15.

[35] Lopez CG, Richtering W (2017). Does flory–rehner theory quantitatively describe the swelling of thermoresponsive microgels?. Soft Matter.

[36] Gao J, Frisken BJ (2003). Cross-linker-free n-isopropylacrylamide gel nanospheres. Langmuir.

[37] Stieger M, Richtering W, Pedersen JS, Lindner P (2004). Small-angle neutron scattering study of structural changes in temperature sensitive microgel colloids. The Journal Chemical Physics.

[38] Kozhunova EY (2018). 1H NMR study of thermo-induced collapse of polyelectrolyte microgels. Express Polym. Lett..

[39] Bird, R. B., Armstrong, R. C. & Hassager, O. *Dynamics of Polymeric Liquids*, Vols 1 and 2 (Wiley, 1987).

[40] Brandrup, J., Immergut, E. & Grulke, E. (eds) *Polymer Handbook*, 4th edition edn (John Wiley, New York, 1999).

[41] Gavrilov AA, Kudryavtsev YV, Chertovich AV (2013). Phase diagrams of block copolymer melts by dissipative particle dynamics simulations. The Journal Chemical Physics.

[42] Flory, P. J. *Principles of Polymer Chemistry* (Cornell University Press, 1953).

[43] Origin software, originlab. https://www.originlab.com/. Accessed: 2019-02-15.

