# Supplementary material for: Towards the realistic computer model of precipitation polymerization microgels
Source: Sci Rep. 2019 Sep 10;9:13052. doi: 10.1038/s41598-019-49512-3 (PMC6737091; doi:10.1038/s41598-019-49512-3)
Supplement: Supplementary file 1 — Supporting Information Towards the realistic computer model of precipitation polymerization microgels [file 41598_2019_49512_MOESM1_ESM.pdf]

Supporting Information
Towards the realistic computer model of precipitation polymerization microgels

Vladimir Yu. Rudyak[1,*], Elena Yu. Kozhunova[1], and Alexander V. Chertovich[2,1]

[1]Lomonosov Moscow State University, Faculty of Physics, Moscow, 119991, Russia
[2]Semenov Institute of Chemical Physics, Moscow, 119991, Russia
[*]vurdizm@gmail.com

Size effects in simulations of microgel synthesis

We simulated precipitation polymerization process for the systems of various sizes. We used simulations box sized of 47^3, 60^3, 75^3, 95^3, 120^3 and 150^3 MD units. All boxes were filled with monomer, cross-linker and initiator particles in ratio of $98.5 : 1 : 0.5$ up to total density of 2.4%. Total numbers of particles were 2.4×10^3, 5.1×10^3, 1.0×10^4, 2.0×10^4, 4.1×10^4, and 8.1×10^4, correspondingly. For systems between 2.4×10^3 and 4.1×10^4 particles, tree independent runs of synthesis process were produced. For the largest system (8.1×10^4 particles), 10 independent runs of synthesis process were produced. All systems were analyzed, and the results were averaged by independent runs for each system size. All error bars represent standard deviations.

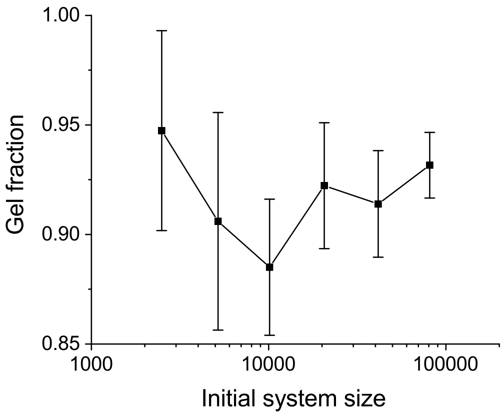

Figure 1. Gel fraction in microgels of various size, conversion rate $c \geq 0.999$.

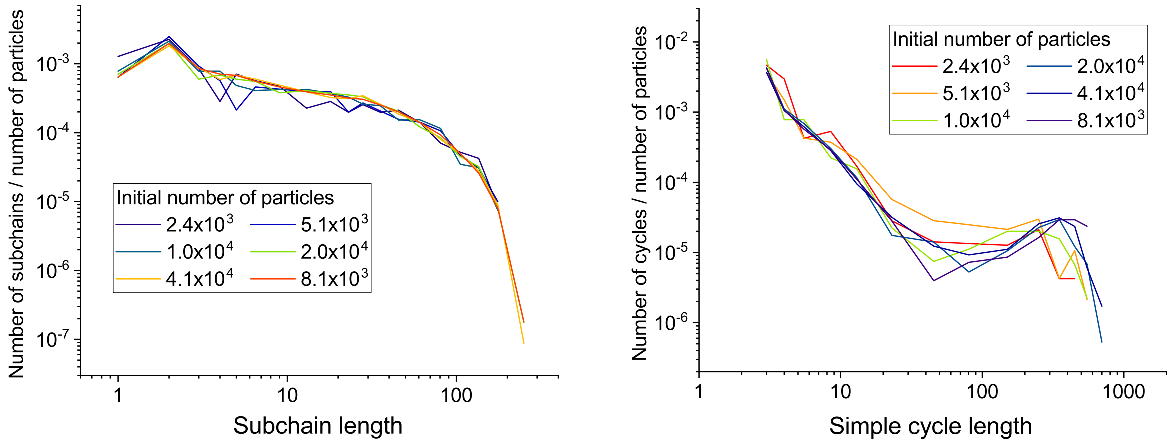

(a) (b)

Figure 2. Topological properties of microgels of various size, conversion rate $c \geq 0.999$.

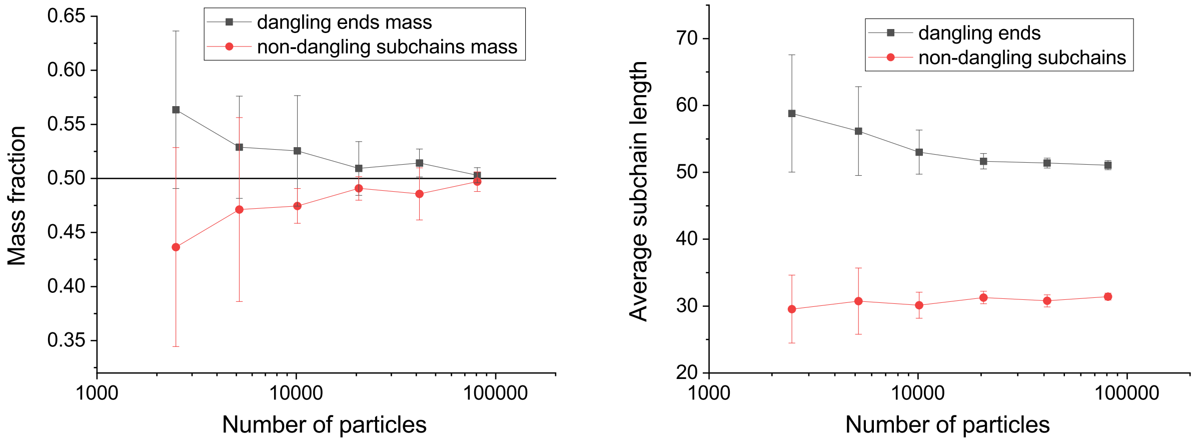

(a) (b)

Figure 3. Dangling ends effects in microgels of various size, conversion rate $c \geq 0.999$.

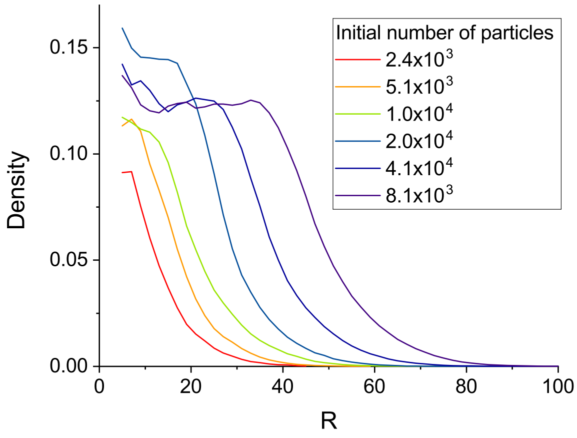

(a)

Figure 4. Density distributions microgels of various size, conversion rate $c \geq 0.999$.

Structure factor of simulated microgel particle during precipitation polymerization process

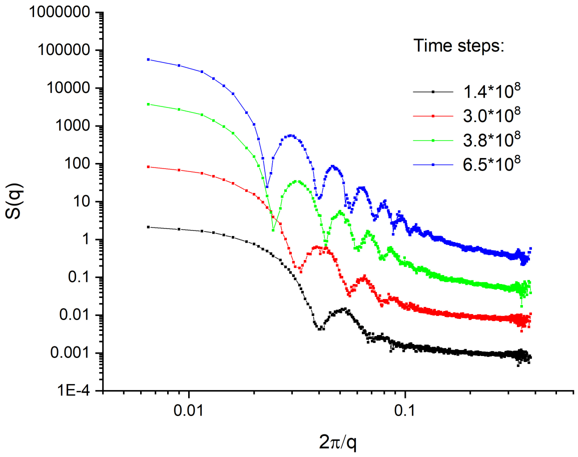

Figure 5. The static structure factor during microgel formation.

Experimental validation of predicted synthesis phases

For validation of predictions based on simulated data, we produced additional analysis of the initial phase of the experimental curing process. For this, we took macrophotographs of the vial with the contrast background every 15 seconds after the beginning of the synthesis (Fig. 6a). We calculated ratio between the physical intensity of white background and black lines (Fig. 6b). During the first 90 seconds of the curing process, the solution remained transparent, and the white-to-black ratio did not change. Then the solution gradually changed to opaque, which corresponds to the fall in white-to-black ratio between 90 and 180 seconds. For two more minutes, the solution opacity increased visually, but the camera was not able to detect it due to the sensor sensitivity limitations. After five minutes, when no more changes could be observed, we stopped the synthesis and started washing of the sample by dialysis. After the dialysis, we analyzed the size of microgel particles in the sample by DLS. In the swollen state (23° C), the primary peak shown the hydrodynamic radius to be equal to approximately 180 nm (compared to 360 nm in fully completed synthesis). Additionally, many small peaks between 5 nm and 25 nm were observed. In the collapsed state (50° C), the measured hydrodynamic radius was about 75 nm. Based on these data, the estimated conversion rate was about 12% (it should be noted this estimation should be compared to the simulated mass fraction of the largest macromolecule instead of the simulated conversion rate, as it counts only for the largest parts of the system).

These data is fully consistent with the results of simulations: gel fraction does not form at all in phase (i), the microgel mass after the phase (iii) is about 5–10% of the maximum microgel mass, and there are many smaller particles are still present in the system at that moment.

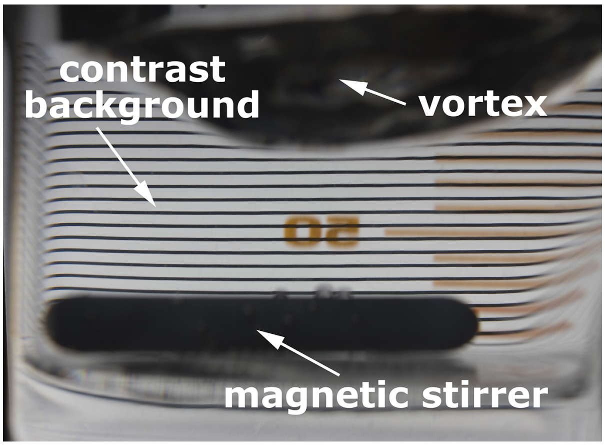

(a)

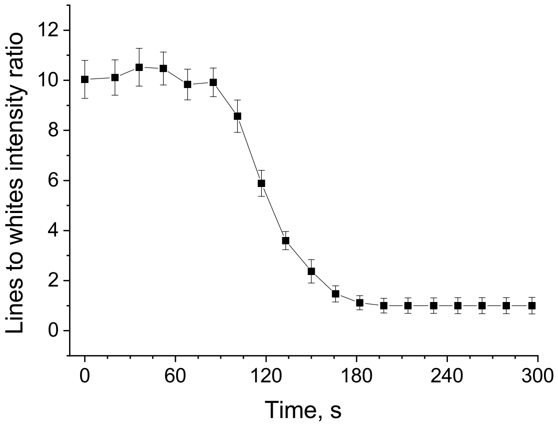

(b)

Figure 6. (a) Experimental setup of transparency measurements during synthesis process. (b) Measured dependency of white-to-black intensity ratio on synthesis time.

Structure factor fitting parameters

Fitting parameters of Flory-Rehner theory applied to the experimental and simulated data of microgel volume fraction ϕ.

Model	Eq. (3)
A	-4.6 ± 0.4
Phi0 (fixed)	0.15
Theta	1.31 ± 0.013
f	0.016 ± 0.005
prefactor (fixed)	2
Reduced Chi-Sqr	3.8^{-4}

Table 1. Fitting parameters for the simulated data.

Model	Eq. (3)
A (fixed)	-9
Phi0 (fixed)	0.15
Theta	32.37 ± 0.17
f	0.06 ± 0.06
prefactor (fixed)	0.35
Reduced Chi-Sqr	0.005

Table 2. Fitting parameters for the experimental data.
